# Dysregulation of BMP, Wnt, and Insulin Signaling in Fragile X Syndrome

**DOI:** 10.3389/fcell.2022.934662

**Published:** 2022-07-06

**Authors:** Chunzhu Song, Kendal Broadie

**Affiliations:** ^1^ Department of Biological Sciences, College of Arts and Science, Vanderbilt University, Nashville, TN, United States; ^2^ Department of Cell and Developmental Biology, School of Medicine, Vanderbilt University, Nashville, TN, United States; ^3^ Kennedy Center for Research on Human Development, Nashville, TN, United States; ^4^ Vanderbilt Brain Institute, School of Medicine, Vanderbilt University and Medical Center, Nashville, TN, United States

**Keywords:** bone morphogenetic protein, insulin-like peptide, fragile x mental retardation protein, wingless, *Drosophila*

## Abstract

*Drosophila* models of neurological disease contribute tremendously to research progress due to the high conservation of human disease genes, the powerful and sophisticated genetic toolkit, and the rapid generation time. Fragile X syndrome (FXS) is the most prevalent heritable cause of intellectual disability and autism spectrum disorders, and the *Drosophila* FXS disease model has been critical for the genetic screening discovery of new intercellular secretion mechanisms. Here, we focus on the roles of three major signaling pathways: BMP, Wnt, and insulin-like peptides. We present *Drosophila* FXS model defects compared to mouse models in stem cells/embryos, the glutamatergic neuromuscular junction (NMJ) synapse model, and the developing adult brain. All three of these secreted signaling pathways are strikingly altered in FXS disease models, giving new mechanistic insights into impaired cellular outcomes and neurological phenotypes. *Drosophila* provides a powerful genetic screening platform to expand understanding of these secretory mechanisms and to test cellular roles in both peripheral and central nervous systems. The studies demonstrate the importance of exploring broad genetic interactions and unexpected regulatory mechanisms. We discuss a number of research avenues to pursue BMP, Wnt, and insulin signaling in future FXS investigations and the development of potential therapeutics.

## Introduction

The *Drosophila* genome contains ∼ 70% conserved homologs of human disease genes, which have been repeatedly proven to mediate equivalent functions in similar cells and tissues (Ugur et al., 2016; Chatterjee and Deng, 2019). A combination of forward and reverse genetic strategies are used to model human diseases (Yamaguchi and Yoshida, 2018). In forward genetics, mutations are randomly induced by chemical mutagens (for example, ethyl methanesulfonate) or transposon insertion (for example, p-elements), with screening for a phenotype of interest. In reverse genetics, targeted mutations are made by transposon-mediated mutagenesis (for example, p-element excision) or, more recently, through using clustered regularly interspaced short palindromic repeats/Cas9 (CRISPR/Cas9). To express or knockdown genes, targetable binary expression systems (for example, Gal4/UAS) allow rescue studies with *Drosophila* or human genes, as well as RNA interference (RNAi) to reduce transcripts at specific times and in defined cells. For neurological disease models, the developing *Drosophila* central nervous system (CNS) has been extensively characterized at the level of individually-identified neural stem cells and neurons (Harding and White, 2018; Rossi et al., 2021). For critical synapse studies, the *Drosophila* glutamatergic neuromuscular junction (NMJ) offers superior imaging and electrophysiological access that has proven invaluable in modeling numerous disease states (Tian et al., 2017; Frank et al., 2020; Tue et al., 2020). Most recently, *Drosophila* brain neural circuit mapping using sophisticated transgenic fluorescent imaging studies and transmission electron microscope ultrastructure reconstruction has provided astonishing single-cell resolution (Golovin et al., 2019; Phelps et al., 2021). Together, these combined tools have allowed *Drosophila* neurological disease modeling to contribute tremendously to fundamental mechanistic discoveries.

*Drosophila* screening approaches have been essential in defining secreted intercellular signaling pathways, including the discovery of Wingless (Wg) as the founding Wnt ligand (Nüsslein-Volhard and Wieschaus, 1980), and the discovery of bone morphogenetic protein (BMP) ligands (Upadhyay et al., 2017). More recent reverse genetic strategies have revealed important roles for *Drosophila* insulin-like peptide (ILP) secretion and signaling (Semaniuk et al., 2021). These secreted signals are critical for numerous cell regulatory processes; including proliferation, differentiation, migration, growth, function, and programmed death (Sedlmeier and Sleeman, 2017; Ng et al., 2019; Saltiel, 2021). At the *Drosophila* glutamatergic NMJ, Wnt/BMP/ILP ligands and their receptors participate in bidirectional *trans*-synaptic neuron-muscle and intercellular neuron-glia communication (Dani et al., 2012; Mahoney et al., 2016; Chou et al., 2020). More generally, interfering with these secreted intercellular signaling pathways in the CNS causes aberrant neurogenesis/gliogenesis, synaptogenesis, and neural circuit remodeling starting in embryonic stages (Luo et al., 2010; Guo et al., 2011), and consequently generating defects in sensory processing, coordinated movement, and higher brain function (Goel et al., 2019; Golovin et al., 2021). Consistently, autistic and neurodegenerative disorders are characterized by poorly regulated secretion of BMPs, Wnts, and ILPs (Timberlake et al., 2017; de Mello et al., 2019; Serafino et al., 2020; Russo and Wharton, 2022). For instance, an Alzheimer’s disease model accumulates Wnt ligands, causing inflammation of postsynaptic cells (Ali et al., 2020). Nevertheless, secreted intercellular signaling in neurological disease states is understudied, especially for neurodevelopment. Recently, *Drosophila* forward and reverse genetic screening strategies have begun to reveal important secretion mechanisms in a disease model context.

Fragile X syndrome (FXS) is the leading neurodevelopmental disorder causing inherited intellectual disability (Razak et al., 2020), often associated with autism spectrum disorder (ASD) comorbidity (Rajaratnam et al., 2017). Most FXS disease cases result from the expansion of CGG repeats (>200) in the 5’ untranslated region of the *Fragile X Mental Retardation 1* (*FMR1*) gene (Hagerman et al., 2017), leading to epigenetic hypermethylation and loss of the gene product Fragile X Mental Retardation Protein (FMRP) (Bagni and Zukin, 2019). A few reported disease cases are point mutations (for example., Gly266GLu (G266E), Ile304Asn (I304N)) in FMRP RNA-binding domains (RBDs), which impair the canonical FMRP mRNA translational regulation function (Starke et al., 2022). Clinically, FXS patients exhibit low-scale IQ, social autism, hyperactivity, and delayed developmental learning/speech (Ciaccio et al., 2017). In mammals, FMRP has two paralogs, Fragile X Related 1 (FXR1) and FXR2, with separable functions (Drozd et al., 2018). Only FMRP loss causes FXS, and only human FMRP can rescue *Drosophila* FXS model neurological defects (Coffee et al., 2010), including supernumerary synapse formation in the NMJ and brain (Pan et al., 2004; Dear et al., 2017), defective brain neural circuit remodeling (Tessier and Broadie, 2008; Doll et al., 2017), and impaired learning/memory (Bolduc et al., 2008; Jiang et al., 2016). Recent studies show BMP, ILP, and Wnt signaling defects are causatively implicated in *Drosophila* FXS disease model phenotypes. This article reviews key discoveries for these secreted intercellular signaling pathways in the *Drosophila* FXS model in comparison with the mouse FXS model and human FXS patients. We discuss promising new avenues for future FXS investigations of signaling defects and the potential for new therapeutic treatment strategies based on the correction of secretory communication impairments.

## Part 1: BMP Signaling in FXS

BMP signaling pathways are widely involved in the regulation of cellular proliferation (Sachdeva et al., 2019), differentiation (Abdal Dayem et al., 2018), and death (Bollum et al., 2017; Yang et al., 2021). Consistently, BMPs have essential roles in neurogenesis and gliogenesis during embryonic CNS development, and these secreted signaling functions are known to be impaired in the FXR family (*FMR1*, *FXR1*, and *FXR2*) mutants. For example, FXR2 deficiency mice exhibit inhibition of BMP signaling through upregulation of the secreted BMP-binding Noggin, which normally functions in preventing BMP ligands from binding to their receptors, resulting in aberrant neural progenitor cell (NPC) proliferation and differentiation within the hippocampal dentate gyrus (DG) (Guo et al., 2011). Acting as an RNA-binding regulator, FXR2 reduces the half-life of the targeted *noggin* mRNA, thereby repressing Noggin protein levels specifically secreted from DG-NPCs and resulting in increased neuronal differentiation and decreased astrocytic differentiation within the developing hippocampus. Both exogenous BMP2 treatment and an endogenous Noggin block in FXR2 knockout mice rescue the neuronal and astrocytic differentiation/proliferation defects of the DG-NPCs (Guo et al., 2011). BMP signaling is also misregulated in human-induced pluripotent stem cells (hiPSCs) obtained from FXS patients (Boland et al., 2017). Gene expression profiling shows that both the BMP7 ligand and the BMP type 2 receptor (BMPR2) are FMRP-target genes in hiPSCs. However, this report stopped short of linking aberrant BMP signaling to defects in neuronal differentiation. It is therefore highly important to investigate the role of BMP ligands and BMPRs in the decision-making mechanisms of stem cells driving neurogenesis/gliogenesis in the FXS condition.

**FIGURE 1:**
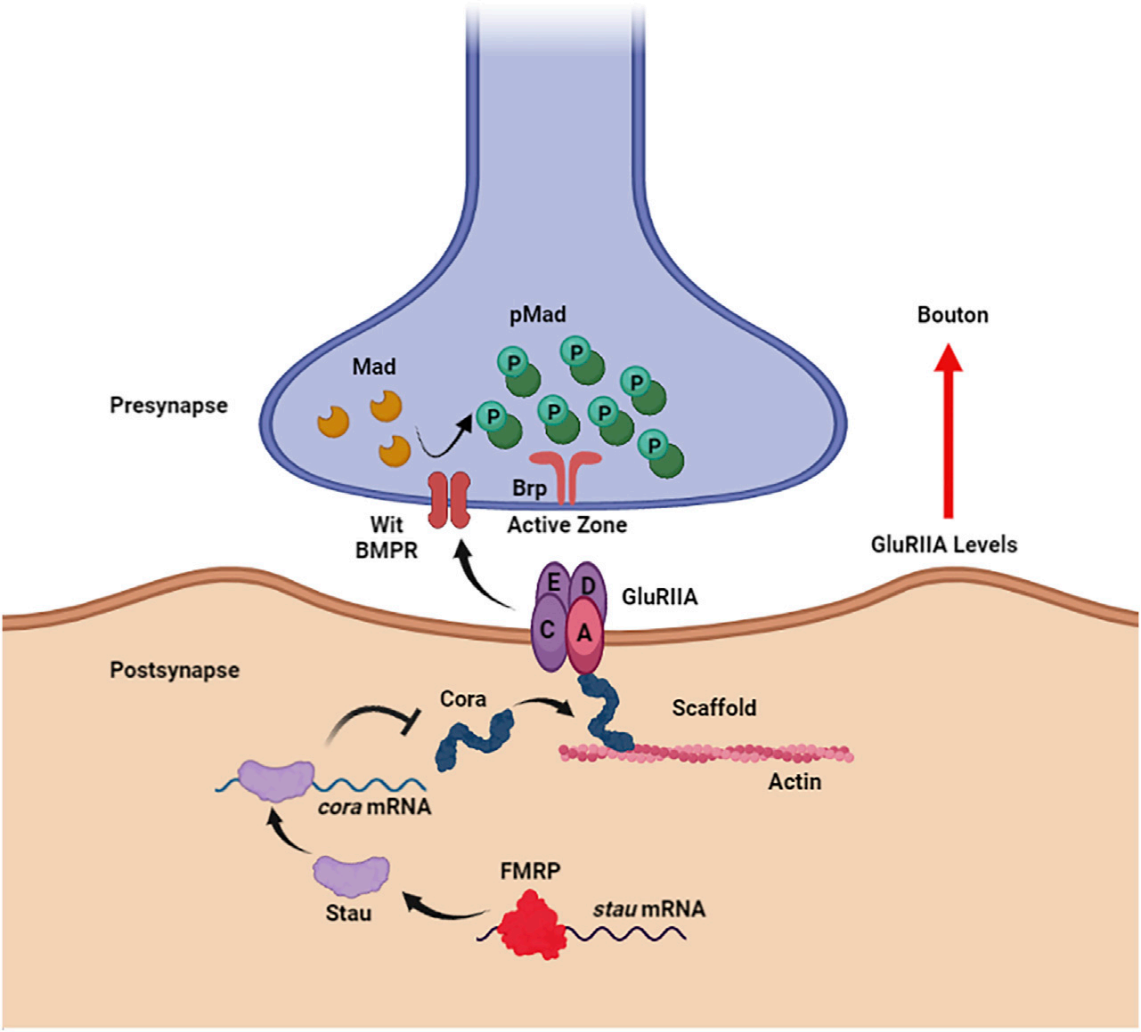
Noncanonical BMP signaling is restricted by FMRP to limit synaptogenesis. In the *Drosophila* larval neuromuscular junction postsynaptic compartment (bottom), FMRP directly binds to *staufen* (*stau*) mRNA to promote translation. Staufen, in turn, binds *coracle* (*cora*) mRNA to inhibit translation. Coracle acts as an actin scaffold to anchor the glutamate receptor type II A (GluRIIA) opposing the presynaptic active zone scaffolded by Bruchpilot (Brp). GluRIIA accumulation induced by loss of postsynaptic FMRP activates noncanonical, *trans*-synaptic signaling via the BMP receptor (BMPR) Wishful Thinking (Wit) to drive Mad phosphorylation (pMad) around presynaptic active zones, resulting in synaptic bouton formation. Figure created with BioRender (BioRender.com).

Later in neurodevelopment, secreted *trans*-synaptic BMP signaling regulates synaptic structure and function at the *Drosophila* larval glutamatergic NMJ (Figure 1), including motoneuron terminal growth (Sulkowski et al., 2016; Kamimura et al., 2019), neurotransmission strength (Kamimura et al., 2019; Politano et al., 2019), and maintained homeostasis (Chou et al., 2020). Three known BMP ligands Decapentaplegic (Dpp), Glass-bottom boat (Gbb), and Screw (Scw) (Upadhyay et al., 2017) are secreted from either presynaptic boutons or postsynaptic muscles to activate BMP type I receptors Thick veins (Tkv) and Saxophone (Sax), and either of two the type II receptors Wishful thinking (Wit) or Punt (Put) (Kim and O’Connor, 2014; Upadhyay et al., 2017). In the presynaptic boutons, BMP signaling promotes microtubule-associated protein (MAP) positive regulator *futsch* (human MAP1B) mRNA translation by repressing mRNA-bound FMRP function, thus up-regulating synaptic growth (Nahm et al., 2013; Kim et al., 2019). In mice, presynaptic FMRP also binds *BMPR2* mRNA (*Drosophila* Wit homolog) to inhibit full-length isoform translation, thus causing accumulation of the noncanonical BMP pathway component Lin11/Isl1/Mec3 domain kinase 1 (LIMK1) within neurons (Kashima et al., 2016). Combining insights from both *Drosophila* and mouse models shows downstream increased LIMK1 hyper-phosphorylates cofilin to stimulate actin polymerization, which, in turn, results in *Drosophila* NMJ bouton and mouse neuronal dendritic spine overgrowth (Kashima et al., 2016), as well as *Drosophila* larval hyperactivity (Kashima et al., 2017). In FXS patient brain cortexes, full-length BMPR2 protein and phospho-cofilin levels are both increased (Kashima et al., 2016), consistent with the *Drosophila* and mouse FXS model discoveries.

In the *Drosophila* NMJ postsynaptic domain, FMRP inhibits noncanonical *trans*-synaptic BMP signaling to negatively regulate presynaptic bouton formation (Figure 1; Song et al., 2022). Postsynaptic FMRP binds double-strand RBP (dsRBP) *staufen* (*stau*) mRNA to stabilize the transcripts in muscle (Figure 1). The translated Stau protein, in turn, binds *coracle* (*cora*) mRNA to restrict translation of this glutamate type II A receptor (GluRIIA) anchoring actin scaffold (Figure 1; (Chen et al., 2005). Coracle belongs to the actin-binding 4.1 ezrin-radixin-moesin (FERM) family, which normally has the receptor-interacting ERM domain on their N-terminus (Chen et al., 2005; McClatchey, 2012). However, the Coracle C-terminus was demonstrated to bind GluRIIA in a yeast two-hybrid study (Chen et al., 2005), therefore the F-actin and glutamate receptor binding domains of Coracle remain ambiguous (Figure 1). Nevertheless, the GluRIIA accumulation in the *Drosophila* FXS model (Pan and Broadie, 2007) is well explained by the postsynaptic FMRP-Stau-Cora regulative pathway, which activates phosphorylation of presynaptic Mothers against Decapentaplegic (Mad) to drive NMJ bouton overgrowth (Figure 1; Song et al., 2022). Interestingly, Coracle overexpression and RNAi phenocopy (Song et al., 2022), as in other neurodevelopmental contexts (Landsverk et al., 2007; Tokuda et al., 2014; Fulterer et al., 2018), and GluRIIA-induced pMad production does not involve BMP ligands (Friedman et al., 2013; Sulkowski et al., 2016; Kamimura et al., 2019), but does depend on BMP receptors Wit and Sax (Sulkowski et al., 2016; Kamimura et al., 2019). GluRIIA is thought to interact with Wit through the transmembrane GluR-clustering protein Neto (Chou et al., 2020), but the mechanism of this FMRP-dependent noncanonical *trans*-synaptic BMP signaling remains to be fully elucidated.

Finally, BMP signaling also has important roles in the regulation of neuronal apoptosis (Hayano et al., 2015) and autophagy (Yang et al., 2021). Since programmed cell death plays key functions in maintaining tissue homeostasis (Ghose and Shaham, 2020), dysregulation of cell death is associated with a variety of human neurodevelopmental diseases, including ASD (Wei et al., 2014; Fricker et al., 2018). In this process, BMP receptors and downstream SMAD (*C. elegans* small (SMA) + *Drosophila* Mad) signaling serve to link mitochondrial and Wnt signaling regulatory networks (see below). Mechanistically, augmented phospho-SMAD1/5/9 (pSMAD1/5/9) binds to the tumor suppresser p53 protein, thus preventing p53 degradation from forming complexes with ubiquitin ligase murine double minute 2 (MDM2) (Hayano et al., 2015). Consequently, accumulated p53 activates the *Bax*-mediated apoptotic pathway in *BMP type 1A receptor* (*BMPR1a*) mutant mice (Hayano et al., 2015). To inhibit autophagy of newborn mice activin A type 1 receptor (ACVR1, another BMP type 1 receptor) mutated cranial neural crest cells (CNCCs), accumulated pSMAD1/5/9 activates mammalian target of rapamycin complex 1 (mTORC1) to block β-catenin degradation and increase Wnt/β-catenin signaling (Yang et al., 2021). While FMRP shows strong interaction with this type of BMP signaling, BMP-mediated neural cell death defects in FXS models have not yet been well studied. Given that RNA-binding FMRP binds to SMAD family transcripts (Ascano et al., 2012), we hypothesize that FMRP controls SMAD protein levels to directly modulate BMP signaling, and this likely impacts the events from neurogenesis to synaptogenesis to the regulation of cell death during neurodevelopment. Taken together, FMRP can directly activate BMP signaling through cascade pathways, and target BMP receptors and downstream molecules, to regulate neuronal development and survival.

## Part 2: Insulin-Like Peptide Signaling in FXS

Studies of aberrant insulin-like peptide (ILP) signaling in FXS originated from elevated phosphatase and tensin (PTEN), target of rapamycin (TOR), phosphoinositide 3-kinase (PI3K), and activated protein kinase B (Akt) in FXS model and patient neurons (Sharma et al., 2010; Hoeffer et al., 2012; Gross et al., 2015), consistent with elevated insulin signaling discovered in from transcriptome profiling of the mouse FXS model hippocampus (Prilutsky et al., 2015). In *Drosophila* FXS stem cells, FMRP also suppresses the insulin-like receptor (InR) *via* LIN-28, an RNA-binding protein required for the translation of insulin-like growth factors (Luhur et al., 2017). *Drosophila* FMRP inhibits ILP secretion from adult brain neurons to enable circadian behavior, and promote short- and long-term memory, through control of downstream PTEN and phospho-Akt (pAkt) activation (Monyak et al., 2017). Genetically reducing ILP ligands or InR in *dfmr1* mutants significantly rescues both the circadian and memory defects, consistent with results of expressing pAkt inhibitor PTEN in *dfmr1* null neurons. In parallel, *dfmr1* mutants fed metformin also show ameliorated short-term and long-term memory defects. Likewise, the mouse FXS model fed metformin shows improved cognitive function and reduced seizure incidence in adults (Gantois et al., 2017). Moreover, metformin treatment also rescues dendritic overgrowth, elevated matrix metalloproteinase 9 (MMP-9) secretion levels, upregulated extracellular-signal-regulated kinase (ERK) signaling, and hyperphosphorylated eukaryotic translation initiation factor 4E (eIF4E) in adult FXS male mice. Consistently, two FXS patients clinically treated with metformin for 1 year showed significant improvement in their cognition and speech behavior (Protic et al., 2019), suggesting that correction of insulin signaling could provide an exciting new avenue for possible FXS therapeutic treatment.

In the *Drosophila* FXS model, FMRP regulation of ILP signaling is involved in CNS development. In *Drosophila* progenitor stem cells (neuroblasts) and subsequently, in developing glia, FMRP sequentially limits the reactivation of larval brain neuroblasts by inhibiting ILP signaling (Callan et al., 2012). Following neuroblast-targeted FMRP knockdown, the number of cells containing cyclin E, a marker of G1/S phase transition, is significantly upregulated only in young animals (6–12 h after larval hatching; ALH), indicating that FMRP is required to restrict autonomous neuroblast reactivation. However, FMRP knockdown specifically in glia elevates cyclin E positive cell number at a later developmental stage (12–24 h ALH), showing FMRP in glia is also required for non-autonomous neuroblast reactivation. Using pAkt as a positive readout for ILP signaling, FMRP loss induces upregulated signaling in neuroblasts, but not in glia (Callan et al., 2012). In developing *Drosophila* adult brains, ILP signaling later participates in neuronal removal when neural circuits undergo remodeling companied by programmed cell death (Chihara et al., 2014; Kessissoglou et al., 2020). To maintain brain homeostasis, glial cells prune neuronal processes and remove entire neurons *via* a phagocytosis mechanism (Freeman, 2015; Kim et al., 2020; Bittern et al., 2021; Raymond et al., 2022). For example in a *Drosophila* adult injury model involving cutting off the antenna, damaged neurons release ILP ligands that activate glial InRs, leading to augmented expression of glial phagocytosis receptor Draper (Drpr) and subsequent glial engulfment and clearance of axons (Musashe et al., 2016). This work clearly shows ILP signaling is involved in the glial phagocytosis of neurons following injury and raises the question of a similar mechanism during normal brain development.

**FIGURE 2:**
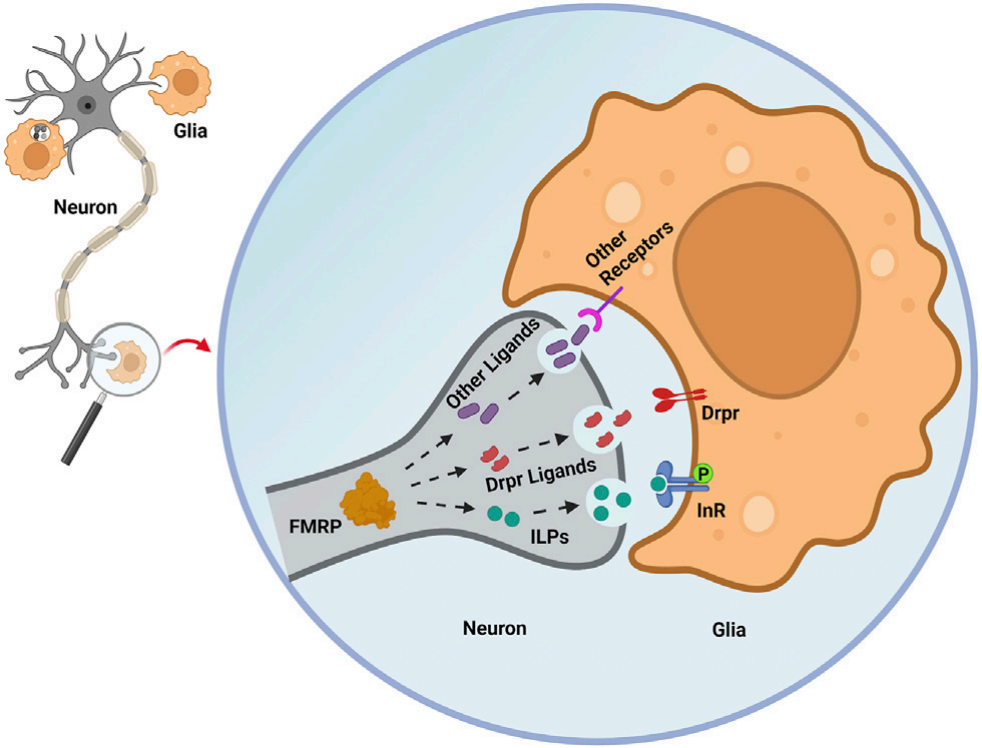
Secreted signals regulated by neuronal FMRP orchestrate glial phagocytosis. In early adult *Drosophila* brain PDF-Tri neurons, FMRP is proposed to promote the secretion of insulin-like peptides (ILPs) that drive glial insulin receptor phosphorylation (InR-P) to trigger glial phagocytosis of neuronal processes. In the glia, Draper (Drpr) phagocytosis receptor expression is decreased by loss of neuronal FMRP. However, the neuronal Drpr ligands (for example, Pretaporter, phosphatidylserine) involved in this FMRP-dependent mechanism remain unknown. Neuronal FMRP may regulate numerous other “find me” and “eat me” secreted neural signals that recruit glia and instruct glial phagocytosis, ranging from individual synapses to whole brain neurons. Figure created with BioRender (BioRender.com).

In developmentally-transient pigment-dispersing factor tri (PDF-tri) clock neurons, FMRP is required to mediate removal from the early adult *Drosophila* brain (Gatto and Broadie, 2011). In the *Drosophila* FXS model, neuron-to-glia ILP signaling is required to drive the Dynamin (*Drosophila shibire*) glial phagocytosis mechanism of neural clearance (Figure 2; Vita et al., 2021). In *dfmr1* mutants, phospho-InR (p-InR) levels are also significantly reduced on glial membranes, correlating with the delayed developmental clearance of the PDF-tri neurons. Furthermore, constitutively activating glial InRs in *dfmr1* mutants restores normal neuron clearance, showing that FMRP-dependent glial InR activation is required for phagocytosis (Figure 2). Moreover, reducing the level of the ESCRT-III membrane remodeler Shrub in *dfmr1* null mutants helps restore the downregulated glial p-InR levels and PDF-tri neuron clearance defects, suggesting FMRP works via Shrub to promote InR phosphorylation and glial phagocytosis (Vita et al., 2021). Note that FMRP regulation of ILP-InR signaling currently appears to be cell-type (for example, neural *vs*. glial InR) and developmental-stage (for example, immature *vs*. mature brain) dependent (Monyak et al., 2017). It is not known how neuronal FMRP-dependent ILP signaling induces glial phagocytosis together with other signals, including neuronally-derived ligands for the glial Draper receptor (Figure 2), which are essential for glial phagocytosis (Vita et al., 2021). FMRP is proposed to regulate the secretion of multiple neuronal ligands, which act sequentially or cooperatively as “find me” and “eat me” signals driving glial phagocytosis (Figure 2). It will be important to identify and order these FMRP-dependent secreted signals and to place them in hierarchical order with ILP to fully understand the glial phagocytosis remodeling mechanism.

## Part 3: Wnt Signaling in FXS

The Wnt acronym derives from *Drosophila* Wingless (Wg), whose developmental role was identified in the famous segment polarity screen (Nüsslein-Volhard and Wieschaus, 1980), and mouse INT-1, from a virus-induced breast tumorigenesis screen (Nusse and Varmus, 1982). There are 7 Wnts in *Drosophila* and 19 in mice. Cysteine-palmitoylated Wnts are secreted with the assistance of seven-pass transmembrane proteins Wntless (Wls) and evenness interrupted (Evi) (Willert et al., 2003; Bänziger et al., 2006; Bartscherer et al., 2006). Wnt receptors include the Frizzled (Fz) family, low-density lipoprotein receptor-related proteins-5/6 (LRP-5/6), receptor tyrosine kinase-like orphan receptor-1/2 (ROR1/2), and related to tyrosine (Y) kinase (Ryk). Importantly, amyloid precursor protein (APP) was recently reported as a receptor for Wnt3a/5a in limiting neural outgrowth in mice (Liu et al., 2021). Wnt signaling pathways are widely involved in developmental decisions, tissue self-renewal, and cell death (Ghosh et al., 2017; Majidinia et al., 2018; Nayak et al., 2018). In FXS disease models, dysregulated Wnt signaling impairs embryonic development, neurogenesis/gliogenesis, and later synaptogenesis. FMRP deficiency causes reduced Wnt signaling, resulting in decreased neuronal differentiation but increased astrocyte differentiation in immature adult neural progenitor cells (aNPCs) in the mouse hippocampus (Luo et al., 2010). FMRP binds glycogen synthase kinase 3β (GSK3β) mRNA, a well-known β-catenin inhibitor in canonical Wnt signaling, with FMRP loss increasing GSK3β levels to inhibit β-catenin in Wnt3a-positive aNPCs. This pathway downregulates neurogenesis and promotes gliogenesis (Luo et al., 2010). This study also reports that FMRP binds cyclin D1 and CDK4 mRNAs to restrict neural progenitor cell proliferation.

In the mouse FXS model, pharmacological inhibition of GSK3β significantly improves hippocampus-dependent learning by rescuing neurogenesis and neuronal maturation defects (Guo et al., 2012), further confirming Wnt signaling is involved in FXS brain development. However, clinical trials of GSK3β inhibition as a potential FXS treatment show only minor improvements (Berry-Kravis et al., 2008; Liu and Smith, 2014; Telias, 2019), possibly because FMRP regulation of Wnt signaling for neural differentiation happens during early development, which was bypassed in these trials (Telias et al., 2015). In the mouse adolescent FXS model, inhibiting GSK3α also corrects aberrant protein synthesis, audiogenic seizures, sensory cortex hyper-excitability, and deficits in learning and memory (McCamphill et al., 2020). In addition to the GSK3 family, several other Wnt/β-catenin signaling pathway component transcripts are also targeted by FMRP in the embryonic mouse cortex, including Abelson Helper Integration Site 1 (Ahi1), Catenin Alpha 2 (Ctnna2), and Catenin Beta 1 (Ctnnb1) (Casingal et al., 2020). The SRY-related HMG-box (SOX) transcription factors modulate Wnt signaling with a variety of mechanisms, including β-catenin interactions and cofactor recruitment (Grimm et al., 2020; Stevanovic et al., 2021). In Wnt signaling, SOX2/9 contributes to neurodevelopment (Liu et al., 2015; Lee et al., 2016; Lefebvre et al., 2019; Kinney et al., 2020) with FMRP inhibiting SOX2 and enhancing SOX9 expression to promote the Fragile X-human neural precursor cell (FX-NPC) neuron-to-glia ratio (Telias et al., 2015). These discoveries suggest Wnt signaling manipulation could be a viable therapeutic strategy for FXS treatment and should motivate researchers to continue screening possible target molecules impacting Wnt signaling.

In the *Drosophila* FXS model, FMRP regulates *trans*-synaptic Wnt signaling to modulate glutamatergic NMJ larval synaptogenesis (Friedman et al., 2013). Wingless (Wg) is the Wnt ligand, although Wnt2/5 could be involved (Liebl et al., 2008, 2010). Frizzled-2 (Fz2) is the Wg receptor. FMRP loss increases Wg secretion from presynaptic boutons to induce cleavage of the larval muscle Fz2 C-terminus (Fz2-C), which is translocated as a second messenger into postsynaptic nuclei (Friedman et al., 2013). Consistently, Wg overexpression within the presynaptic motor neuron will activate Fz2-C accumulation within postsynaptic muscle nuclei (Mathew et al., 2005). In the *Drosophila* FXS model, the GPI-anchored heparan sulfate proteoglycan (HSPG) glypican Dally-like protein (Dlp) acting as a Wg co-receptor, as well as the transmembrane HSPG syndecan (Sdc), are both highly elevated at the NMJ synaptic terminal (Friedman et al., 2013). In *dfmr1* mutants, elevated presynaptic Wg secretion and postsynaptic Dlp co-receptor levels drive larval supernumerary synaptic bouton formation and elevated neurotransmission strength. Genetically restoring Dlp and Sdc in the *dfmr1* null independently rescues the NMJ structure and function defects (Friedman et al., 2013). As Dlp is negatively regulated by secreted heparan sulfate 6-O-endosulfatase (Sulf1) to promote Fz2-C signaling (Dani et al., 2012), the reduced nuclear Fz2-C level in *dfmr1* mutants suggests that FMRP may be required to restrict Dlp by maintaining Sulf1, thus increasing Fz2-C translocation to postsynaptic nuclei. Taken together, FMRP acts at multiple levels to regulate Wg *trans*-synaptic signaling, including presynaptic Wg secretion, postsynaptic Wg co-receptor control, and the signal transduction of the cleaved Fz2-C receptor second messenger into the postsynaptic nuclei.

In the *Drosophila* FXS model, FMRP regulates the secretion of matrix metalloproteinase 1 (MMP1), a proteinase that cleaves extracellular proteins, to modulate larval synaptic structure and function by modulating secreted Wnt signaling (Dear et al., 2016, 2017). Null *dfmr1* mutants exhibit upregulation of MMP1 and MMP1 proteolytic enzymatic activity surrounding presynaptic boutons at the glutamatergic NMJ. *Drosophila* only has two MMPs (secreted MMP1 and the GPI-anchored MMP2), with the secreted protease specifically affected by FMRP. In the mouse adult FXS model, secreted MMP7/9 are likewise positively upregulated out of at least 23 MMPs, correlated with Wnt signaling differences (Ingraham et al., 2011; He et al., 2012). Whereas direct studies of a Wnt-MMP regulatory network have not been reported in mice, the synaptic MMP1 upregulation *dfmr1* larval mutants is prevented by genetically correcting synaptic Dlp levels (Dear et al., 2017). The mechanism works downstream of neuronal activity to control rapid synaptic bouton formation, with Dlp promoting the localized synaptic MMP1 proteolytic activity. These findings indicate an FMRP-Wg-Dlp-MMP1 regulatory network interacts in the secreted synaptomatrix space to control activity-dependent NMJ synaptogenesis. One hint at the mechanism is that MMP2 cleaves Dlp in the *Drosophila* ovary so that it no longer acts as a Wg co-receptor (Wang and Page-McCaw, 2014). It can therefore be hypothesized that MMP2-dependent Dlp processing may antagonize the MMP1-Dlp interaction, consequently resulting in less postsynaptic Fz2-C translocation in the FXS condition. While the mechanism needs further investigation, these studies demonstrate Wnt signaling dysregulation in FXS synaptogenesis, providing novel directions to pursue possible treatments.

## Conclusion and Future Directions

### FMRP-Dependent BMP/ILP/Wnt Signaling at the Synapse

This article reviews and discusses BMP, ILP and Wnt secreted signaling dysfunction in Fragile X syndrome (FXS) in different developmental stages, particularly in the nervous system. New discoveries suggest that FMRP occupies core roles linking BMP, ILP, and Wnt regulatory networks that mediate neurogenesis, gliogenesis, synaptogenesis, and glial functions during neural circuit remodeling. To study synaptogenesis and neurotransmission function regulated by this FMRP-dependent signaling, the *Drosophila* glutamatergic NMJ provides an attractive model to test ligand secretion, receptor activation, co-receptor function, and the downstream second messenger cascades. For BMP signaling, a new noncanonical *trans*-synaptic pathway involves postsynaptic FMRP and Staufen RNA-binding proteins regulating the FERM Coracle scaffold for glutamate receptors communicating through presynaptic BMP receptors to activate local Mad phosphorylation (p-Mad) and drive synaptic bouton formation (Song et al., 2022). This novel FMRP-Staufen-Coracle-GluRIIA-BMPR-pMad pathway strengthens neurotransmission (Figure 1). While the mechanism of bouton development is limited by an FMRP-BMPR-LIMK1-cofilin pathway in the presynaptic terminal is relatively well studied (Kashima et al., 2016), we do not know how postsynaptic FMRP induces *trans*-synaptic signaling *via* BMP receptors. Although there is good evidence that pMad accumulates around presynaptic active zones with the removal of postsynaptic FMRP (Song et al., 2022), the mechanism by which pMad is induced and works with other interactors to regulate synaptogenesis remains to be studied. Since pMad is well-known to work with the cofactor Medea (Med) to serve as a transcription factor (Berndt et al., 2020), it would be interesting to map gene expression related to synaptic development modulated by presynaptic pMad-Med interaction following targeted postsynaptic knockdown of FMRP.

Numerous studies show BMP signaling bidirectionally interacts with insulin signaling to modulate cell metabolism, growth, and programmed death (Clark et al., 2021; Kim and O’Connor, 2021; Mao et al., 2021). In *Drosophila* motor neurons, insulin signaling also negatively regulates presynaptic neurotransmitter release via the FOXO-dependent regulation of the eukaryotic initiation factor 4e binding protein (4eBP) translational inhibitor (Mahoney et al., 2016). It will be important to test if presynaptic FMRP acts upstream of the PTEN-PI3K-pAkt-FOXO pathway to control this functional secretion mechanism. FMRP was also just recently reported to modulate activity-dependent bulk endocytosis (ADBE) in the mouse FXS model (Bonnycastle et al., 2022), suggesting the need for further testing of synaptic vesicle cycling and trafficking mechanisms. On the postsynaptic side of the larval *Drosophila* NMJ, InR-mediated signaling induces synaptic development through the guanine-nucleotide exchange factor dPix promoting Discs Large (Dlg) scaffold recruitment to the muscle subsynaptic reticulum (SSR) (Ho and Treisman, 2020). With the new evidence that postsynaptic FMRP restricts presynaptic bouton formation (Figure 1), it is possible that a postsynaptic FMRP-BMP-ILP network regulates NMJ growth expansion through secreted ligands and receptor activation. Broadening this interaction even further, NMJ Wnt (Wg) signaling in the FXS disease model is likely also connected to this network. In *dfmr1* mutants, presynaptic Wg secretion is elevated (Friedman et al., 2013), but we do not yet know if this increased secretion is regulated by presynaptic FMRP, postsynaptic FMRP, or possibly both. While postsynaptic FMRP is suspected to participate in cleaved Fz2-C intracellular translocation or degradation, this involvement is still speculative. Continuing to explore the FMRP-dependent control of *trans*-synaptic signaling mechanisms remains a high priority.

### FMRP-Dependent BMP/ILP/Wnt Signaling in Neuron-to-Glia Communication

Multiple FMRP-dependent secreted signals likely mediate intercellular communication between neurons and glia in brain development and circuit remodeling (Figure 2). In the CNS, BMPs modulate neuronal metabolism (Xu et al., 2017; Jensen et al., 2021), synaptic plasticity (Vickers et al., 2020; Jensen et al., 2021), blood-brain barrier function (Wevers and de Vries, 2016; Abdullahi et al., 2017a, 2017b; Petersen et al., 2021), and cell death (Hart et al., 2020). Mouse BMP2-10 are broadly distributed across the brain, while BMP11-15 has not been well studied (Jensen et al., 2021). None of the *Drosophila* BMP ligands (Dpp, Gbb, Scw) have been well characterized in the CNS, but likely show a similarly robust distribution. In both mouse and *Drosophila*, multiple reports demonstrate that BMP signaling plays essential roles in communication between neurons and glia in development, in neural remodeling after injury, and during aging (Petersen et al., 2017; Díaz-Moreno et al., 2018; Sasaki et al., 2019; Hart et al., 2020). However, the role of BMP signaling in the CNS of FXS disease models has not yet been investigated. In the *Drosophila* FXS model, FMRP is required in neurons, but not glia, for glial phagocytosis during brain clock circuit remodeling (Figure 2; Vita et al., 2021). FMRP binds *Drosophila* BMPR2 homolog Wit mRNA to inhibit translation (Kashima et al., 2016), suggesting it might limit BMP signaling on the receptor side. FMRP may also regulate secreted “find me” or “eat me” signals to affect neuronal removal during adult brain circuit remodeling (Figure 2). In this hypothesis, is it possible that glial-secreted BMPs feedback to neuronal FMRP-modulated phagocytosis signals? In addition, loss of FMRP increases pMad signaling in neurons (Song et al., 2022), and reduced neuronal pMad may impair glial-dependent neuronal clearance in the FXS model. It will be important to test possible FMRP-pMad mechanisms of glial phagocytosis.

The role of FMRP-dependent insulin and Wnt signaling in neuron-glia communication has just begun to be studied. Neurally secreted ILPs are suspected to activate glial engulfment for neuronal clearance (Vita et al., 2021). However, it is not clear if the secreted ILP signal is a “find me” or “eat me” signal activating the glial InRs (Figure 2). *Drosophila* InRs in ensheathing glia and astrocyte-like glia are required for neuronal clearance following injury (Musashe et al., 2016) as well as restriction of lifespan extension (Woodling et al., 2020). In mice, an InR deficiency in astrocyte glia leads to aberrant morphology, mitochondrial function, and circuit connectivity (García-Cáceres et al., 2016; Rhea and Banks, 2019). In *Drosophila*, signaling downstream of activated glial InRs promotes Akt phosphorylation, which is essential for Draper phagocytosis receptor expression (Musashe et al., 2016). These discoveries provide exciting hints that neuronal FMRP may facilitate ILP secretion to activate glial phagocytosis function by promoting Draper expression, which could also activate glia to respond to the neuronal FMRP-controlled “eat me” signals (Figure 2). It will be important to integrate roles of proposed neuronal “eat me” ligands activating Draper, such as phosphatidylserine (PS) and Pretaporter (Kuraishi et al., 2009; Kurematsu et al., 2022). Moreover, Wnt signaling may also likely play a role in neuron-glial communication in the FXS model (Casingal et al., 2020; Peteri et al., 2021). Perhaps we can learn about neuron-glia Wnt signaling from neurodegeneration disease models? For example, mouse Parkinson’s disease (PD) models show the Wnt/β-catenin pathway plays a central role in the response of astrocyte and microglia to neuroinflammation, neural mitochondrial dysfunction, dopaminergic neuroprotection, and oxidative stress (Marchetti, 2020). Similarly, it will be important to test the possible role of the neuron-glial Wnt (Wg) signaling cascade in the FXS model. We need to explore how Wnt signaling may work together with secreted BMP and ILP signals to regulate glial phagocytosis during brain circuit remodeling. Numerous studies suggest that both Wnt and BMP signaling bidirectionally regulate an insulin-dependent network for developmental homeostasis (Chen et al., 2010; Foley, 2012; Cabrae et al., 2020; Baboota et al., 2021; Mao et al., 2021; Tian et al., 2021). BMP-Wnt cross interactions also help maintain physiological processes (Thorne et al., 2018; Chhabra et al., 2019; Wang et al., 2020). Nevertheless, it is hard to define upstream and downstream roles in this signaling. Although FMRP has a number of direct targets in all three signaling cascades, it is urgent to profile the overlapping core targets to guide drug design for FXS animal models and clinical trials. Identifying therapeutic treatments is desperately needed to combat devastating neurological impairments in FXS patients.
