# Corrigendum: Dysregulation of BMP, Wnt, and insulin signaling in fragile X syndrome

**DOI:** 10.3389/fcell.2024.1416720

**Published:** 2024-06-04

**Authors:** Chunzhu Song, Kendal Broadie

**Affiliations:** ^1^ Department of Biological Sciences, College of Arts and Science, Vanderbilt University, Nashville, TN, United States; ^2^ Department of Cell and Developmental Biology, School of Medicine, Vanderbilt University, Nashville, TN, United States; ^3^ Kennedy Center for Research on Human Development, Nashville, TN, United States; ^4^ Vanderbilt Brain Institute, School of Medicine, Vanderbilt University and Medical Center, Nashville, TN, United States

**Keywords:** bone morphogenetic protein, insulin-like peptide, fragile x mental retardation protein, wingless, *Drosophila*

In the published article, there was an error in the legend of **Figure 2** as published. The expression level of glial Draper with loss of neuronal FMRP was mistakenly summarized. Based on the conclusion of our reviewed paper, loss of neuronal FMRP should decrease the expression level of glial Draper. The corrected **Figure 2** legend appears below:

Figure 2 | Secreted signals regulated by neuronal FMRP orchestrate glial phagocytosis. In early adult *Drosophila* brain PDF-Tri neurons, FMRP is proposed to promote the secretion of insulin-like peptides (ILPs) that drive glial insulin receptor phosphorylation (InR-P) to trigger glial phagocytosis of neuronal processes. In the glia, Draper (Drpr) phagocytosis receptor expression is decreased by loss of neuronal FMRP. However, the neuronal Drpr ligands (for example, Pretaporter, phosphatidylserine) involved in this FMRP-dependent mechanism remain unknown. Neuronal FMRP may regulate numerous other “find me” and “eat me” secreted neural signals that recruit glia and instruct glial phagocytosis, ranging from individual synapses to whole brain neurons.

The authors apologize for this error and state that this does not change the scientific conclusions of the article in any way. The original article has been updated.

